# 3D culture system of muscle precursor cell to reveal mechanosensing defects in nuclear envelope related disorders

**DOI:** 10.1186/1750-1172-10-S2-O4

**Published:** 2015-11-11

**Authors:** Catherine Coirault

**Affiliations:** 1 Sorbonne Universités, UPMC Univ Paris 06, INSERM UMRS974, CNRS FRE3617, Center for Research in Myology, Paris, France

## 

Mutations in the LInker of the Nucleoskeleton and Cytoskeleton (LINC)-complex associated proteins, including lamins and nesprins cause human muscular dystrophies but disease mechanisms still remain to be elucidated. We aim to determine whether human muscular dystrophies resulting from mutations in A-type lamin and nesprin1 affected the capacity of myoblasts to sense the stiffness of the extracellular matrix. Human myoblasts with various mutations in the A-type lamins encoded by *LMNA* (*LMNA*), nesprin1 mutant (*SYNE1*), and control (WT) myoblasts were cultured in 3D soft matrix (1-10 kPa) or on 2D conventional glass (~ 10^6^ kPa) surfaces. Focal adhesion (vinculin), actin cytoskeleton, and YAP signaling pathway, a particularly important regulator of the mechano-response, were investigated. On 2D hard surface, there was no obvious difference in actin cytoskeleton and focal adhesion between WT, LMNA and SYNE1 myoblasts. In contrast, LMNA and SYNE1 myoblasts cultured in soft matrix exhibited enlarged focal adhesions and stress fibers compared with WT. Cytoplasmic translocation of YAP observed in WT in response to reduced stiffness matrix was absent in LMNA and SYNE1 cells, suggesting a permanent activation of YAP in mutant cells. In conclusion, our data indicate that cell culture matrix stiffness is critical to reveal mechanosensing defects in dystrophic muscle cells.

